# Human Respiratory and Gut Microbiomes—Do They Really Contribute to Respiratory Health?

**DOI:** 10.3389/fped.2020.00528

**Published:** 2020-09-03

**Authors:** Juliana Durack, Claus T. Christophersen

**Affiliations:** ^1^Symbiome Inc., San Francisco, CA, United States; ^2^Division of Gastroenterology, Department of Medicine, University of California, San Francisco, San Francisco, CA, United States; ^3^School of Medical and Health Sciences, Edith Cowan University, Joondalup, WA, Australia; ^4^WA Human Microbiome Collaboration Centre, School of Molecular and Life Sciences, Curtin University, Perth, WA, Australia

**Keywords:** gut microbiome, microbiome development, asthma, acute respiratory disease, respiratory microbiome

## Abstract

Human gastrointestinal and respiratory tracts are colonized by diverse polymicrobial communities shortly after birth, which are continuously molded by environmental exposure. The development of the resident microbiota in early life is a critical factor in the maturation of a healthy immune system. Disturbances to the intricate relationship between environmental exposure and maturation of the infant microbiome have been increasingly identified as a potential contributor to a range of childhood diseases. This review details recent evidence that implicates the contribution of gut and airway microbiome to pediatric respiratory health.

## Introduction

The human superorganism has coevolved with a wide variety of microbial species that inhabit the human body in an assortment of body-site-specific consortia (1–4). These resident microbes support many functions in the human body, including the metabolism of complex carbohydrates. Fermentation of carbohydrates leads to, amongst other products, anti-inflammatory, anti-proliferative short-chain fatty acids (SCFAs) that represent an essential energy source for gastrointestinal (GI) epithelial cells (5–7). Other microbial-derived bioactive metabolites include essential vitamins (8, 9), hormones (10, 11), and neurotransmitters (12, 13). Resident microbes also modulate drug absorption (14) and the efficacy of vaccines (15).

Of equal importance is the role of the microbiome in protection against pathogenic organisms via competitive colonization (16–18), in addition to microbial regulation of the development and subsequent modulation of local and systemic, innate and adaptive immune function (19–21). This is particularly applicable to the evolving early-life microbiome, which has been recognized as having a strong influence on long-term health from childhood through to adulthood (22).

The early-life human microbiota is evident shortly after birth at multiple body sites, and it continues to assemble, evolve and mature throughout childhood, continuously shaped by environmental exposures. Early exposures to environmental toxicants, livestock, and pet ownership, along with birth delivery by cesarean section (vs. vaginal birth), exclusive formula (rather than breast) feeding, and antimicrobial administration, have all been implicated in shaping the developing microbiome in children (23–29). Disturbances to the intricate relationship between environmental exposure and the maturation of the microbiome have been increasingly identified as an enhancer in the development of a range of childhood diseases including those affecting the airways (30–34). This review aims to summarize the most recent and compelling evidence that implicates the contribution of gut and airway microbiome in modulating pediatric respiratory health. The focus of this review is on acute respiratory illness, recurrent wheeze, and asthma, with the omission of cystic fibrosis, which has recently been reviewed (35–37).

## The Origin of Gut and Airway Microbiota at Birth

Although there is emerging evidence of perinatal microbial exposure, based on the identification of microbial communities in the peri- (38) and post-natal meconium (39–41), the vast proportion of microbial colonization of the human body takes place postpartum. According to studies examining infant microbiome development at multiple body sites, colonization with niche-specific microbial assemblages is evident in the first few weeks following delivery (26, 40). Pioneer microbial colonizers of the infant gut have been tracked to multiple maternal body sites in vaginally delivered infants, with strains originating from the maternal gut accounting for a substantial proportion of the overall microbial abundance in the newborn gut, followed by maternal vaginal, oral and skin microbial reservoirs (40). The gut microbiome of infants delivered by Cesarean section was mostly devoid of the maternal gut commensals, particularly of the genus *Bacteroides* (27, 42), and was more likely to include maternal skin and oral microbes, as well as bacteria found in the delivery room (42–45).

Significantly less is known about the origin of the initial airway microbial colonizers, as no studies to-date have mapped a direct species transfer from the mother to the infant. However, the presence of microbial species common to multiple maternal body sites has been observed in the oral microbiota of newborns (40), with these pioneer communities also influenced by the delivery mode (46). Since the microbiota of the lungs is thought to originate primarily from the dispersion of microbes from the oral, and to a much lesser extent the nasal cavity (2, 3, 47), the former likely serves as the route of initial microbial transmission for the lower airways at birth. What is certain, however, is that the nascent microbiome, both in the intestinal and airway tracts continues to acquire microbes from maternal sources and their surrounding environment including other family members and caretakers in the first few years of life (40, 48). Maternal health, reflected in the composition of her microbiome, in addition to other environmental factors, may be necessary for the appropriate assembly of nascent microbial communities in infancy. Improving our understanding of which key microbial species are required during this early developmental window for the geneses of health would aid the ongoing efforts of developing efficacious microbiota-targeted therapeutics as a potential preventative strategy in mitigating the onset of disease. This is an enormous task and, without a doubt, the *Holy Grail* of pediatric microbiome research to date.

## Microbiome Maturation—the Influence of Environment Over Genetics and What it Means for Respiratory Health

The microbial composition is unique for each individual irrespective of body site, even in the first few days of life. These nascent communities continue to diversify shaped by environmental exposure as the gut and airway microbiota assemble and mature (39, 49, 50). The infant microbial ecosystem assembly follows body-site specific yet coordinated trajectories (51), resulting in distinct microbial biogeography at each body site, with a personalized unique microbial fingerprint for each individual (52).

The uniqueness of individual microbial assemblies could be partly attributable to host genetics, with several studies linking genetics to microbiota composition in a healthy population (53, 54). Other more recent studies of >1,000 participants found the impact of genetics on the microbiota to be less influential (55, 56), pointing to environmental factors as the main contributors to inter-person microbiome variability in healthy individuals. Although genetically related individuals tend to harbor more similar microbiota (27, 57, 58), this relative similarity is likely due to shared environmental exposures and cohabitation, which facilitates microbial transfer between family members (59, 60). This concept is supported by an observation of gut microbiome divergence in monozygotic twins after living apart (60).

Environmental exposures appear to play a pivotal role in the early microbial acquisition and community succession in infancy. Exposures such as the aforementioned cesarean section delivery (27, 29, 42, 44, 61–63), peri- and post-natal antibiotic use (64, 65), gestational age (66), maternal health (26, 39, 67, 68), and early diet (42, 62, 63, 67), have all been associated with variability observed in the composition and successional trajectories of the gut microbiota in the early years. A recent study assessing the effect of delivery mode on the gut microbiota maturation of 120 newborns in the first year of life, independent of intrapartum antibiotics, showed enrichment of *Bifidobacterium* and a reduction of *Enterococcus* and *Klebsiella spp*. in vaginally delivered infants, who exhibited a more stable microbiota development (29). Mode of delivery, infant feeding, and antibiotic use have also been shown to alter the upper respiratory microbiota maturation in infancy (49, 69). Infants born by vaginal birth were quicker to acquire *Corynebacterium* and *Dolosigranulum spp*. in their nasopharyngeal microbiota than their caesarian-born counterparts. The acquisition of these species coincided with enhanced microbial community stability and fewer number of respiratory tract infections in the first year of life (69, 70).

The environment surrounding the infant serves as a natural microbial source that can colonize different body sites and potentially modulate immune maturation and tolerance. Oral (23) or intranasal (71) installation of house-dust from low-risk for asthma households protected mice against airway allergen challenge in experimental models of asthma. Gut microbiota of mice gavaged with house dust from homes with dogs was enriched for *Lactobacillus johnsonii* (23). Oral supplementation with this species reduced allergic airway sensitization to cockroach allergen, reducing airway levels of pro-inflammatory cytokines IL-4, IL-5, and IL-13 (23). This response was mediated by enhanced levels of immunomodulatory fatty acids, including docosahexaenoic acid (72). Similarly, nasal administration of farm-derived bacteria, either *Lactococcus lactis, Acinetobacter lwoffii* (73), or *Staphylococcus sciuri* (74), resulted in decreased hallmarks of T helper type 2 (Th2) driven allergic airway inflammation. Results from these mouse-model-based experiments are consistent with the observation that children who grow up with dogs or are exposed to farms and livestock are less likely to develop atopy and childhood asthma (75) and have distinct microbiota in infancy (24, 76–78). However, it should be noted that only a limited number of microorganisms from the environment are ecologically adapted for the successful and persistent colonization of a mammalian host. Colonizing germ-free mice with microorganisms present in diverse environmental samples resulted in a small fraction of these surviving in the mouse gut. Most of the surviving organisms were replaced by more adapted mouse or human-derived microbial strains (79).

The acquisition and appropriate development of the infant microbiome appear to be important in establishing a healthy host-microbiome symbiosis, and disruption of this harmonious relationship has been associated with childhood respiratory diseases. For instance, delayed gut microbiota maturation (39, 68) and decreased microbial diversity (80, 81) in the first year of life has been observed in infants at higher risk for asthma development. Whereas, disruption of microbial niche specificity along the respiratory tract (2, 3), together with an influx of oral commensals in the nasopharynx, appears to precede the development of respiratory tract infections (82). An improved understanding of the intricate interplay between early life microbiota acquisition, environmental microbial engraftment, and immune conditioning is needed to elucidate the microbial impact on the etiology of childhood respiratory disease.

## Microbiota Development and the Immune System

Microbial assembly in infancy appears to be vital in establishing appropriate local and systemic innate and adaptive immune functions (83). A recent study elegantly demonstrated that exposure to a specific subset of intestinal microbiota enriched in riboflavin-synthesizing bacteria in the first few weeks of life is necessary for appropriate mucosal-associated invariant T (MAIT) cells development in the skin, lungs and small intestine of mice (84). Conversely, colonization later in life failed to promote MAIT cell development within tissues, indicating that exposure to specific microbes must occur during an early-life window for correct immune priming.

The long-term effect of the appropriate microbial priming of the host on immune system development is particularly evident from studies of germ-free (GF) mice. These animals have a significantly underdeveloped immune system with a lack of regulatory gut CD4+CD8αα+double-positive intraepithelial T lymphocytes (85), possess fewer T regulatory cells (86), display a thinner gut (87), and lung (88) mucus lining and have reduced expression of immunoglobulin A (89) necessary for clearance of pathogenic microbes from the gut lumen (90). Similar immune dysregulation is also observed in neonatal mice following exposure to broad-spectrum antibiotics (91, 92). Neonatal mice exposed to a single dose of macrolide antibiotic soon after birth exhibited persistent alterations in their gut microbiota composition, ileal gene expression, intestinal T-cell populations and significant reduction of fecal IgA levels. These features were not observed in neonatal GF or conventional adult mice receiving the same treatment (91). Absence of microbial colonization of the lungs appears to have a less profound effect on the subset of localized immune cells (88), except iNKT cells which are present at higher levels in both lungs and gut of GF mice (93) and dissipate following microbiota-transfer from conventional (non-GF) animals.

Exposure of neonatal mice to broad-spectrum antibiotics is known to diminish microbial diversity and leads to an exacerbation of allergen-induced airway inflammation (94, 95). Specifically, antibiotic treatment reduced the number of SCFA producing gut bacteria, leading to a subsequent reduction in systemic levels of immunomodulatory SCFAs (5, 95, 96). Conversely, supplementation of mice with SCFA or increasing dietary fiber ameliorated allergic airway inflammation in sensitized animals (5, 95). This resulted in reprogramming of hematopoiesis and subsequent seeding of the lungs by dendritic cells with high phagocytic capacity but an impaired ability to promote Th2 cell effector function or to transport inhaled aeroallergens to lung draining nodes. Airway microbiota has also been shown to influence the development of allergic airway inflammation. Immediately after birth, neonatal mice exhibit enhanced Th2 cell inflammation and airway hyperresponsiveness following exposure to house dust mite aeroallergens. However, as the airway microbiome matured, the exacerbated response to aeroallergen diminished (97). The absence of airway colonization during this early postpartum window resulted in sustained susceptibility to allergic inflammation through adulthood. Supporting these murine observations are pediatric epidemiological studies focused on the etiology of childhood disease, which implicate early-life antibiotic administration as a risk factor for childhood respiratory disease development (98, 99). Collectively, these observations provide strong evidence for the establishment of commensal microbial communities early in life as a critical factor for healthy immune development, ensuring appropriate control, and a potential reduction in cases of airway inflammation.

## Airway Microbiome and Respiratory Health

Evidence implicating perturbations to the composition and function of the airway microbiota in pediatric respiratory disease has grown substantially in the last decade (30, 31, 34, 100). Unlike studies of the enteric microbiota, which are primarily based on profiling the highly abundant fecal microbial communities, studies of the airway microbiota present more of a challenge.

### Challenges Associated With Studying the Airway Microbiome

Challenges of studying the airway microbiome arise from a relatively low microbial burden typically recovered from airway samples and the distinct composition of microbes present in samples collected using different methods and the respiratory tract compartment being sampled, making it difficult to compare key findings between independent studies. The airway extends from the nasal opening to the alveoli of the lungs, with each compartment providing a distinct microenvironment for microbial colonization. Ideally, samples for microbiome studies addressing associations with pulmonary disease should be obtained at a site where the inflammatory processes contributing to respiratory symptoms occur, and for most pulmonary conditions, these manifest in the lower airways. Unfortunately, sampling the lower airways, particularly in children who cannot expectorate, requires invasive bronchoscopy. This procedure is poorly suited for studies involving infants, healthy children, and large-scale studies due to the need for anesthesia and specialized procedural expertise. As a result, extensive studies of lower airway microbiome in young children are lacking. Most clinical studies focused on the airway microbiota in children are based on the non-invasive samples of the upper respiratory tract (e.g., nasopharyngeal/nasal swabs and nasal wash/lavage); these will form the main focus of discussion in this review.

The upper airway is a poor surrogate for the lower airway microbiota (2, 3, 47, 101–104). Compared to the lower airways, the upper airways (both nasal and nasopharyngeal compartments) harbor less complex microbiota, comprising of distinct microbial communities (3, 47, 103, 104). Microbial composition of the lower airway is more reflective of the oral microbiota (2, 3, 47, 105, 106). However, it should be noted that a loss of microbial topography along the respiratory tract have been observed in individuals with respiratory disease (3, 107), with nasal microbiota contributing a portion of respiratory disease-associated bacterial taxa to the lower airways (3, 47, 103). The exact mechanisms of interaction between the upper and lower airway microbiota in respiratory disease remain unclear. However, there is growing evidence for microbe-microbe interactions and microbe-host interactions (3, 104) within and across various compartments of the respiratory tract.

Dissimilar microbiota was also observed between sampling methods within the same compartment of the respiratory tract, such as those described in bronchial brush vs. bronchial alveolar lavage (108), and nasal brush vs. nasal wash samples (109). This is also the case for the two most representative anatomical sites of the upper airway microbiome studies, the nares and nasopharynx, which comprise of distinct microbial assemblies with considerable compositional overlap (110). Interpretation of airway microbiota studies should, therefore, be carried out with caution, delineating not only the different compartments of the respiratory tract but also based on sample collection procedure. Despite the aforementioned limitations, there has been tremendous recent progress in uncovering the role of the developing upper airway microbiome in modulating and improving respiratory health.

### Upper Airway Microbiome and Acute Respiratory Illness

**Figure 1:**
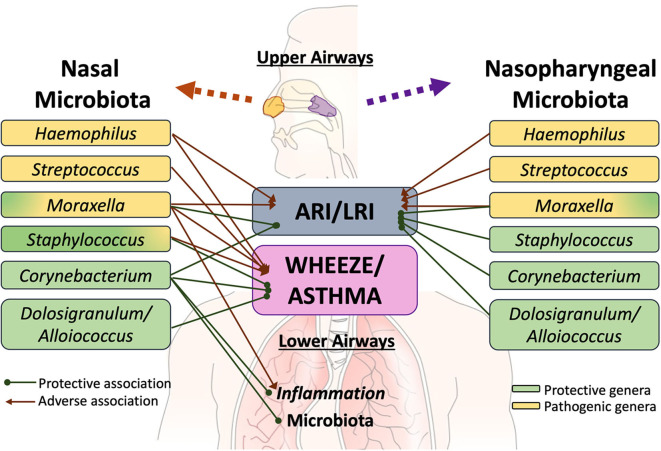
Specific members of the upper airway microbiota have been differentially linked to acute respiratory illness (ARI), including lower respiratory illness (LRI), recurrent wheeze, asthma risk and exacerbation. Although limited, there is some evidence that interactions exist between at least some members of the upper airway microbiota with those colonizing the lower airways and with the pulmonary immune system. However, further work is required to elucidate these links.

Several extensive studies have characterized the upper respiratory tract microbiome in children as being most frequently dominated by one of the following six bacterial genera—*Moraxella, Streptococcus, Corynebacterium, Staphylococcus, Haemophilus*, and *Alloiococcus* [annotated as *Dolosigranulum* in some databases] (50, 107, 111–114). These distinct bacterial microbiota profiles differentially relate to respiratory illness (Figure 1). In a study of 234 children at high risk for atopy, early colonization of the nasopharynx with *Haemophilus, Streptococcus* or *Moraxella* was found to be strongly associated with acute respiratory illness (ARI), including lower respiratory illness (LRI) in the first 5 years of life (50). Conversely, the relative abundance of *Staphylococcus, Corynebacterium*, and *Alloiococcus* in the upper airways of infants under 2 years of age were negatively associated with ARIs. The incidence rate of LRIs was highest in children with an early nasal *Moraxella*-dominant profile and lowest in those with a *Corynebacterium*-dominant microbiota profile (113). In a different matched case-control study of 307 children hospitalized with LRIs and 154 age-matched controls, nasopharyngeal microbiota profiles dominated by *Haemophilus influenzae* and *Streptococcus pneumoniae* were significantly associated with LRIs whereas those dominated by *Moraxella catarrhalis/nonliquefaciens* or by *Corynebacterium propinquum* and *Dolosigranulum pigrum* were related to relatively stable health (107). Although most ARI events involved viral pathogens, shifts in the microbiota toward dominance by one of the pathogenic bacterial genera preceded the detection of viral pathogens and acute respiratory symptoms (50). Risk of severe respiratory tract illness was significantly increased when rhinovirus (RV) or respiratory syncytial virus (RSV) were detected concurrently with nasal *Moraxella, Streptococcus*, or *Haemophilus* (111, 112, 115, 116). Similarly, in children with LRIs caused by respiratory syncytial virus (RSV), the relative abundance of *H. influenzae* and *S. pneumoniae* in the nasopharynx was strongly associated with increased inflammation (117–119) characterized by overexpression of genes linked to neutrophil/macrophage activation and signaling (117). Young adults with nasal *Moraxella*-dominated microbiome cluster exhibited the most increase in the concentration of inflammatory markers and the highest viral load during experimental RV infection (120). Conversely, among children hospitalized for bronchiolitis, those harboring *Haemophilus* dominant nasopharyngeal and nasal microbiota had increased odds for intensive care treatment and an extended hospital stay, compared to those with *Moraxella*-dominated microbiome profiles (110, 121). Together these observations suggest that bacterial colonization may increase susceptibility to and amplify the host innate immune response to viral respiratory pathogens, thus modulating the severity of ARIs.

### Upper Airway Microbiome in Children With Recurrent Wheeze and Asthma

The same three opportunistic respiratory pathogens (i.e., *Streptococcus*; *Haemophilus*, and *Moraxella*) have also been implicated in increasing the risk for recurrent wheeze (50, 111), asthma development and exacerbation during childhood (69, 115, 122). In a recent study of 842 infants hospitalized for bronchiolitis, increased nasal *Moraxella* and *Streptococcus* relative abundance was associated with a higher risk of recurrent wheezing by age 3 years (123). In another study of 413 school children with asthma, those that experienced at least one exacerbation irrespective of RV infection were more likely to possess *Moraxella*-dominated nasal microbiota composition (112) and elevated eosinophil cationic protein concentration in nasal secretions (a marker for activated eosinophils)—suggesting that such microbial communities may promote asthma exacerbation in the absence of RV. In support of this were *in vitro* observations of enhanced epithelial damage and gene expression of the pro-inflammatory cytokines IL-8 and IL-33 following exposure of human alveolar epithelial cells to cell-free products of nasal *Moraxella* isolates. Additionally, *Moraxella*-dominated nasal communities were found to be more stable over time, and children who persistently exhibited this microbial signature in their longitudinal samples were more likely to have viral asthma exacerbations and a higher number of ARIs (112). Relatedly, the relative abundance of nasal *Moraxella* was positively correlated with systemic and lower airway eosinophilia and bronchial pro-inflammatory cytokine levels in adult subjects with asthma (3). Conversely, children with *Alloiococcus, Corynebacterium*, and *Staphylococcus*-dominated nasal microbiota had decreased risk of respiratory virus detection and asthma exacerbation (112). These findings agree with a similar study of 214 children with asthma, in whom nasal microbiota co-dominated by *Corynebacterium* and *Dolosigranulum* was associated with better asthma control with fewer exacerbations (114) than those with nasal microbiota dominated by more pathogenic bacteria (i.e., *Moraxella, Staphylococcus*- specifically *S. aureus*-and *Streptococcus*). Additionally, loss of asthma control was accompanied by a shift in nasal bacterial communities from *Corynebacterium*/*Dolosigranulum*-dominated to those dominated by *Moraxella*. This transition was associated with the highest risk of exacerbation compared to the other combinations (114). During periods of uncontrolled asthma, the relative abundance of nasal *Corynebacterium* was associated with a lower risk of progression to severe exacerbation, suggesting that members of this genera may modulate airway inflammation in asthma (114). This appears to be the case in adult asthmatics whose nasal microbiota are less frequently dominated by members of this genus compared to healthy controls and in whom levels of bronchial inflammation inversely associated with the relative abundance of nasal *Corynebacterium* (3). Asthmatic subjects with *Corynebacterium*-dominated nasal microbiota exhibited a lower relative abundance of asthma-associated bronchial microbial taxa (3), possibly explaining reduced inflammation observed in these subjects. Similarly, the relative abundance of nasal *Corynebacterium* associated with lower transcription of inflammatory gene expression in the nares of children (104) and showed the highest associations with bronchial genera compared to other nasal taxa. Collectively these observations highlight the need and importance to better understand the fluctuation of microbial assemblies in the upper airways overtime and mechanisms by which these contribute to childhood respiratory disease.

### A Synopsis on the Key Upper Airway Bacterial Players in Respiratory Health

Overall there is consensus on a beneficial role of *Corynebacterium, Alloiococcus/Dolosigranulum*, and coagulase-negative *Staphylococcus* (CoNS; i.e., other than *S. aureus*) species in moderating airway inflammation. Most studies summarized above agree that the loss of these commensals is associated with enhanced pro-inflammatory immune activation. Competitive colonization is a plausible way by which upper airway commensals protect against pathogen colonization and overgrowth. For instance, *Corynebacterium* has been shown to inhibit the growth of *S. pneumoniae* (124) and *S. aureus* (125, 126) by releasing the antibacterial free fatty acids that may prevent nasal colonization with the pathogenic organisms. Similarly, nasal commensal CoNS *S. epidermidis* triggers antimicrobial peptide production in the nasal epithelium, providing it a competitive advantage over *S. aureus* and *M. catarrhalis* leading to decreased inflammation *in vivo* (127). Additionally, *S. epidermidis* has been shown to enhance interferon-λ-dependent immunity against viral influenza resulting in the suppression of viral replication in the nasal mucosa (128). Collectively these observations indicate that from an ecological perspective, a microbiome at equilibrium may resist colonization with pathogenic bacteria and that this microbial stability is important for the maintenance of a healthy airway.

However, when it comes to pathogenic bacteria and associations with respiratory disease, there are currently a lot of unknowns, particularly regarding *Moraxella*. Understanding species and strain-specific differences between *Moraxella spp*., colonizing distinct compartments of the airway, by incorporating more granular approaches such as metagenomics into future studies will likely reveal whether more pathogenic strains are prevalent in certain cohorts. A strain-specific granularity in airway microbiome studies would also uncover whether certain pathogenic types of *Moraxella spp*., are able to descend deeper into the respiratory tract or remain localized in the nasopharynx. The co-occurrence of microorganisms also needs to be considered in future studies of the respiratory microbiota since microbes do not exist in isolation but in complex communities where members influence the virulence potential of opportunistic pathogens. This is seen with co-occurring *Moraxella* which attenuated the positive association of *Alloiococcus* with ARI in older children (50); the observation of *Moraxella* co-occurring with other members of this genus in asthmatic children likely enhancing their virulence potential (112); or the distinct dynamics of associations reported between microbial members in the upper airways of asthmatic and healthy children colonized with the same dominant microbial genera (104). Such studies may help to explain the discrepancy concerning members of genus *Moraxella* observed in pediatric studies summarized above. Improving our understanding of the microbe-microbe associations within and across the airway compartments and relating them to microbe-host mucosal and systemic immune interactions in multidisciplinary studies will help uncover the mechanisms by which opportunistic respiratory pathogens contribute to various respiratory illnesses in children.

## Gut Microbiome and Respiratory Health

The gut is the most densely colonized organ of the human body harboring a diverse range of microbial symbionts, including bacteria, archaea, protozoa, and fungi (129–133). In recent years the importance of the bidirectional crosstalk along the gut-lung axis has been increasingly recognized as a contributor to respiratory health, although the mechanisms of these interactions remain poorly elucidated (134, 135). Unsurprisingly, respiratory diseases are often accompanied by gastrointestinal (GI) comorbidities and *vice versa*. Patients with obstructive pulmonary disease, for example, have increased intestinal permeability during severe acute exacerbations (136) and are 2–3 times more likely to be diagnosed with irritable bowel disease (137), whereas impaired pulmonary function is prevalent in patients with chronic GI disease (138–140). In infants, *Bacteroides*-dominated fecal microbiota was associated with a higher likelihood of being hospitalized for bronchiolitis (141). Although this does not directly support the role of the microbiome in the gut-lung crosstalk, the associative evidence is strong.

Further evidence for the gut-lung axis crosstalk comes from studies implicating early-life gut microbiome perturbations, characterized by loss of commensal *Bifidobacteria, Lachnospira, Faecalibacteria*, and *Akkermansia* (39, 68, 80, 81) in risk for childhood asthma development. Loss of these enteric commensals was accompanied by depletion of fecal acetate, anti-inflammatory polyunsaturated fatty acids and breast milk oligosaccharides, all known to influence gut epithelial colonization of infants at high risk for asthma (39, 80, 81). Mice born to dames gavaged with feces from high-risk infants exhibited exacerbated allergic lung inflammation in an experimental model of asthma, which was alleviated upon the supplementation of dames with bacterial taxa depleted in the microbiota of high-risk infants (80). This suggests a causal role of these early-life enteric bacteria in preventing airway inflammation. Furthermore, soluble products of the gut microbiota from high-risk infants induced Th2 cell expansion, increased IL-4 expression, and decreased regulatory T cell populations *ex vivo*, the latter attributed in part to elevated fecal levels of the oxylipin 12,13-diHOME (81). This oxylipin was initially observed in airways of adult asthmatics following a bronchial challenge with birch pollen (142). The link between pulmonary inflammation and elevated fecal levels of 12,13-diHOME was found to be related to reprogrammed dendritic cell activity and a reduction in the number of pulmonary Treg cells (143). In the infant gut, three epoxide hydrolase genes (EH), responsible for the production of the lipokine were encoded by *Bifidobacterium bifidum* and *Enterococcus faecalis*. Either elevated concentration of 12,13-diHOME or increased abundance of the EH genes was found to significantly increase the probability of developing childhood allergies, eczema, and asthma (143). Future research is needed to determine the role of the gut environment in the expression of these bacterial genes and how it relates to increased pulmonary inflammation in asthma. This will be important as *B. bifidum* is a widely used probiotic for infants, and certain strains encoding EHs may not be suitable for all infants or conditions. It should also be noted that 12,13-diHOME has a profound role in brown adipose tissue activation and is negatively correlated with body-mass index and insulin resistance in mice (144). It remains unclear why this lipokine seems to have different effects in distinct mammalian tissues/systems and what role the time of exposure to elevated levels plays in the development of various pathologies.

### Gut Metabolome and Infant Diet

Infant diet is the earliest, well-established microbial selection pressure, where exclusive breastfeeding is known to select for distinct microbiota compared to formula-feeding (145). Breastfeeding has also shown to mitigate against LRI in a study of 5,322 children in whom breastfeeding for 6 months was associated with a lower incidence of LRI up to 4 years compared to children who had never been breastfed (146). The timing of solid food introduction and its nutritional composition have also been shown to significantly alter the gut microbiome in infants (147). The impact of different dietary habits on shaping the developing gut microbiota has been demonstrated in a comparative study of European and African children aged 1–6 years (148). A significantly higher intake of fiber with low animal protein and fat consumption in the rural African diet promoted enrichment of *Prevotella* and *Xylanibacter* compared to that of *Bacteroides* observed in the western children, leading to significantly increased levels of bacteria-derived fecal anti-inflammatory SCFA (148, 149). The microbial capability of SCFA production by fermenting complex carbohydrates is also evident in infants before weaning (150, 151), highlighting the potential of the infant gut microbiome to ferment complex carbohydrates beyond inulin, fructo- and galacto-oligosaccharides currently used in infant formula.

Harvesting enteric microbial capacity to produce SCFAs via nutritional intervention represents an attractive avenue for gut microbial modulation as a preventative strategy for respiratory disease development (152). As demonstrated in several studies, SCFAs promote intestinal-epithelial integrity leading to reduced inflammation locally in the gut as well as in the respiratory tract (5, 95, 153–155). The mechanisms linking microbial-derived SCFAs and effects on the respiratory tract are just beginning to be elucidated; however, there is no doubt that this relationship is multifaceted (135). Mechanisms of SCFAs mode of action on modulating immune responses have been linked to G protein-coupled receptors and inhibition of histone deacetylase activity (135, 154). It is essential to keep in mind that SCFA effects are not only dependent on their availability, concentration and affinity to receptors but also on the expression of various transporter molecules and downstream effectors in distinct cell types (135). Whether dietary interventions aimed at modulating gut microbiota prove effective in preventing respiratory disease in children remains to be determined.

### Gut Interventions to Improve Respiratory Health

To date, although some promising results have been seen from trials using symbiotics (probiotics/prebiotics or a combination) in reducing the rate of pediatric respiratory tract infections (156, 157), this had not been the case for attempts at preventing atopic asthma (158–161). Encouragingly, infant gut microbiota composition, metabolic function, and host-immune interaction have been shown to be susceptible to modulation by a single *Lactobacillus rhamnosus* strain administered to high risk for asthma infants from birth once-daily for 6 months (39). The positive effect of the probiotic on the microbiome was not sustained following cessation of supplementation, suggesting the need for earlier intervention or use of a multispecies probiotic supplement consisting of species more adapted to the neonatal gut environment to achieve long term-term efficacy (32, 39). Overall, evidence implicating gut microbial alterations in respiratory disease development is rapidly building. More mechanistic studies are needed to improve the understanding of underlying mechanisms driving microbe-microbe and microbe-host interactions locally in the gut in parallel with these along the gut-lung axis before targeted interventions will likely be shown to have clinical efficacy.

## Summary and Concluding Remarks

Convincing evidence from both murine and human studies implicates perturbations to the composition and function of airway and gut microbiota in pediatric respiratory disease. Disruptions to the developmental assembly of the microbiota maturation have long-lasting consequences manifesting in an enhanced response to viral or allergen exposure and consequently, respiratory disease. Despite tremendous progress in uncovering underlying microbial mechanisms responsible for respiratory disease development or exacerbation, the microbiome field is in a nascent state, and many knowledge gaps and opportunities for improved understanding remain. An integrative systems biology approach linking all the members of the microbiota (bacteria, fungal and viral) in the respiratory and gastrointestinal compartments to host immune function is required to elucidate specific microbial mechanisms that govern respiratory disease susceptibility.

## Author Contributions

JD and CC conceived and wrote the review article. All authors contributed to the article and approved the submitted version.

## Conflict of Interest

JD is employed by a skincare company Symbiome Inc. The remaining author declares that the research was conducted in the absence of any commercial or financial relationships that could be construed as a potential conflict of interest.
